# Applications and Challenges of Auditory Brain‐Computer Interfaces in Objective Auditory Assessments for Pediatric Cochlear Implants

**DOI:** 10.1002/EXP.20240078

**Published:** 2025-03-06

**Authors:** Qi Zheng, Yubo Wu, Jianing Zhu, Kunyun Feng, Yanru Bai, Guoping Li, Guangjian Ni

**Affiliations:** ^1^ Academy of Medical Engineering and Translational Medicine Tianjin University Tianjin China; ^2^ Haihe Laboratory of Brain‐Computer Interaction and Human‐Machine Integration Tianjin China; ^3^ State Key Laboratory of Advanced Medical Materials and Devices Tianjin University Tianjin China; ^4^ Centre for China‐UK Audiology Education Institute of Sound and Vibration Research University of Southampton Southampton UK

**Keywords:** auditory brain‐computer interface, auditory cortex remodeling, cochlear implant, cross‐modal reorganization, objective auditory assessment

## Abstract

Cochlear implants (CI) are the premier intervention for individuals with severe to profound hearing impairment. Worldwide, an estimated 600,000 individuals have enhanced their hearing through cochlear implantation, with nearly half being children. The evaluations after implantation are crucial for appropriate clinical interventions and care. Current clinical practice lacks methods to assess the recovery of advanced auditory functions in cochlear‐implanted children. Yet, recent advancements in electroencephalographic (EEG) techniques show promise in accurately evaluating auditory rehabilitation in this demographic. This review elucidates the evolution of brain‐computer interface (BCI) technology for auditory assessment, focusing primarily on its application in pediatric cochlear implant recipients. Emphasis is placed on promising clinical biomarkers for auditory rehabilitation and the neural adaptability accompanying cortical adjustments after implantation. Additionally, we discuss emerging challenges and prospects in applying BCI technology to these children.

## Graphical abstract

Auditory brain‐computer interface connects the brain with external devices and decodes neural signals related to auditory perception. Time‐domain and frequency‐domain analyses are commonly used to identify biomarkers associated with auditory rehabilitation, such as cortical auditory evoked potentials and mismatch negativity. Additionally, source and functional connectivity analyses are employed to elucidate the process of auditory function reconstruction post‐implantation from the perspective of cortical neural plasticity.

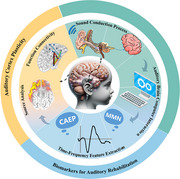

## 1 Introduction

Hearing impairment is a pervasive health issue globally. The 《World Report on Hearing》 indicates that, of the estimated global population of 4.6 billion, an estimated 13% (approximately 610 million individuals) suffer from moderate to severe auditory impairment [1]. Projected statistics for 2050 suggest an alarming increase, with nearly 2.5 billion people anticipated to experience some level of hearing deficits, among which a minimum of 700 million will necessitate hearing assessments and rehabilitative interventions [2]. The implications of hearing loss for patients and their social networks are profound, inducing multiple layers of life stress, particularly in children and adolescents [3]. Consequently, the significance of in‐depth comprehension and effective responses to the challenges of hearing impairment demands critical emphasis.

CIs are the most effective treatment for severe to profound hearing loss [4]. Approximately 600,000 individuals worldwide have improved their hearing abilities through CIs, about 50% of whom are children [5]. CIs operate by directly stimulating auditory nerves with electric pulses to facilitate auditory input to the brain [6, 7]. However, due to inherent technical and physiological constraints, they cannot fully replicate normal biological hearing. The CI's sound processing system is designed to convert acoustic signals into electric signals. However, owing to limitations in voltage and time, this system generally provides information only of limited frequency and intensity [8]. Most existing CIs employ a spectral peak encoding strategy [9], which extracts spectral peak information from the acoustic signal and then converts these data into electric signals [7]. Another widely used coding strategy is continuous interleaved sampling [10]. The main innovation of this strategy is incorporating temporal fine‐structure encoding into the electrical stimulation pattern [11]. Nevertheless, this strategy does not involve preserving natural temporal information between sound signals, which affects the user's performance in processing music and complex auditory environments [12, 13]. Furthermore, due to the limitations of human cochlear size and electrode design, commercially available CIs typically contain only 12 to 22 electrodes. In contrast, a normal cochlea contains approximately 3500 inner hair cells and around 12,000 to 15,000 outer hair cells [14], capable of distinguishing thousands of different frequencies. In order to mitigate interference between electrodes, a specific distance must be maintained between each one. This requirement may lead to the incomplete conversion of auditory information in certain low‐ and high‐frequency regions [15]. Consequently, the number of CI electrodes also restricts its frequency resolution performance. These factors contribute to the inadequacies of CI users in noisy environments, in understanding tonal languages, and in enjoying music compared to those with normal hearing.

Hence, auditory evaluations administered post‐cochlear implantation are pivotal for timely clinical interventions and adjustment [16]. More significantly, the results from these auditory assessments offer vital information that guides healthcare professionals in developing personalized treatment plans [17]. Moreover, the outcomes from regular post‐operative auditory assessments reflect the changes and improvements in hearing restoration after implantation [18].

In a clinical context, the auditory abilities of individuals with CIs are assessed through subjective and objective assessments. Audiologists more frequently employ subjective methods such as questionnaire assessments, pure tone audiometry, and speech assessments, which involve gathering extensive data over a short period and observing daily hearing situations [19, 20, 21]. These methods help assess auditory and sentence recognition abilities in quiet or noisy environments. They require participants to subjectively express heard sound signals or audiologists to make subjective judgments based on the participants' auditory behavior. When these methods are applied to CI children, various factors may easily influence the results, leading to inaccuracies. These factors include parents' educational level, intellectual capabilities, and environmental and physical conditions [22, 23, 24]. Existing clinical objective auditory assessments encompass methods such as auditory brainstem response (ABR), otoacoustic emissions (OAE), electrically evoked auditory brainstem response (E‐ABR), and neural response telemetry, which are primarily used for pre‐operative and intra‐operative evaluations and pertain to the assessment of peripheral auditory system function [25]. Consequently, there is a pressing clinical need for objective assessment methods to evaluate central auditory processing functions.

The auditory brain‐computer interface (ABCI) is suitable for objectively evaluating central auditory processing functions. It establishes a direct communication pathway between users and computers, utilizes electroencephalography (EEG) to record brain electrical activity, and enables researchers and clinicians to observe the brain's response to auditory stimuli directly [26]. This direct observation of neural activity offers valuable insights into auditory processing mechanisms. Moreover, compared to other techniques such as magnetoencephalography (MEG), functional magnetic resonance imaging (fMRI), and positron emission tomography (PET), ABCI holds advantages in terms of its compatibility with CIs, portability, and lower costs [27]. These traits potentially make ABCI the most suitable means for examining neural responses in CI users. Consequently, in recent years, ABCI technology has been extensively applied in the research and assessment of advanced auditory functions [28].

The inception of ABCI evaluations in advanced auditory functionalities dates back to the mid‐twentieth century. In the 1960s, scholars initiated observations of alterations in cerebral electrical activity when human subjects encountered auditory stimuli, leading to the identification and description of certain responses known as cortical auditory evoked potentials (CAEP) [29]. Preliminary investigations primarily focused on basic sound detection and the effects of sound intensity variations on cerebral responses [30]. Throughout the late 1970s and early 1980s, the mismatch negativity (MMN), an automated response to unforeseen auditory events, was discovered [31]. The emergence of MMN signified a notable advancement in utilizing EEG evaluations for research in advanced auditory processing. As of the 1990s, ABCI studies expanded to include more intricate auditory tasks such as music and language processing. Evoked potentials of increased complexity, such as the P300, began receiving extensive attention in exploring cognitive mechanisms within advanced auditory processing [32]. At the onset of the 21st century, propelled by advancements in EEG technology and analytical methodologies, scholars probed deeper into the auditory processing realms, encompassing facets like brain networks and functional connectivity [29]. Since 2010, ABCI techniques have gradually been used to diagnose and evaluate conditions like hearing impairments and language disorders [33, 34]. Today, ABCI objective evaluations have transitioned from laboratory environments to real‐world applications, equipping professionals with potent tools for auditory rehabilitation assessments and interventions for CI users.

The ability to accurately analyze the activities of sophisticated auditory functions and their processing within the brain via ABCI continues to pose significant scientific challenges. Identifying biomarkers capable of reflecting the alterations in auditory functionalities among CI users emerges as a predominant challenge [35]. Post‐cochlear implantation, increased auditory input leads to modifications in the auditory cortex, demonstrating marked plasticity [36]. The precise assessment and interpretation of such plasticity is a substantive issue facing ABCI today. Notably, auditory functionalities are not solitary achievements of the auditory cortex but involve the collaborative operation of several brain networks [37]. Capturing and deciphering interactions and synchronizations between these intricate networks utilizing EEG continues to represent a significant hurdle needing development [38].

**FIGURE 1:**
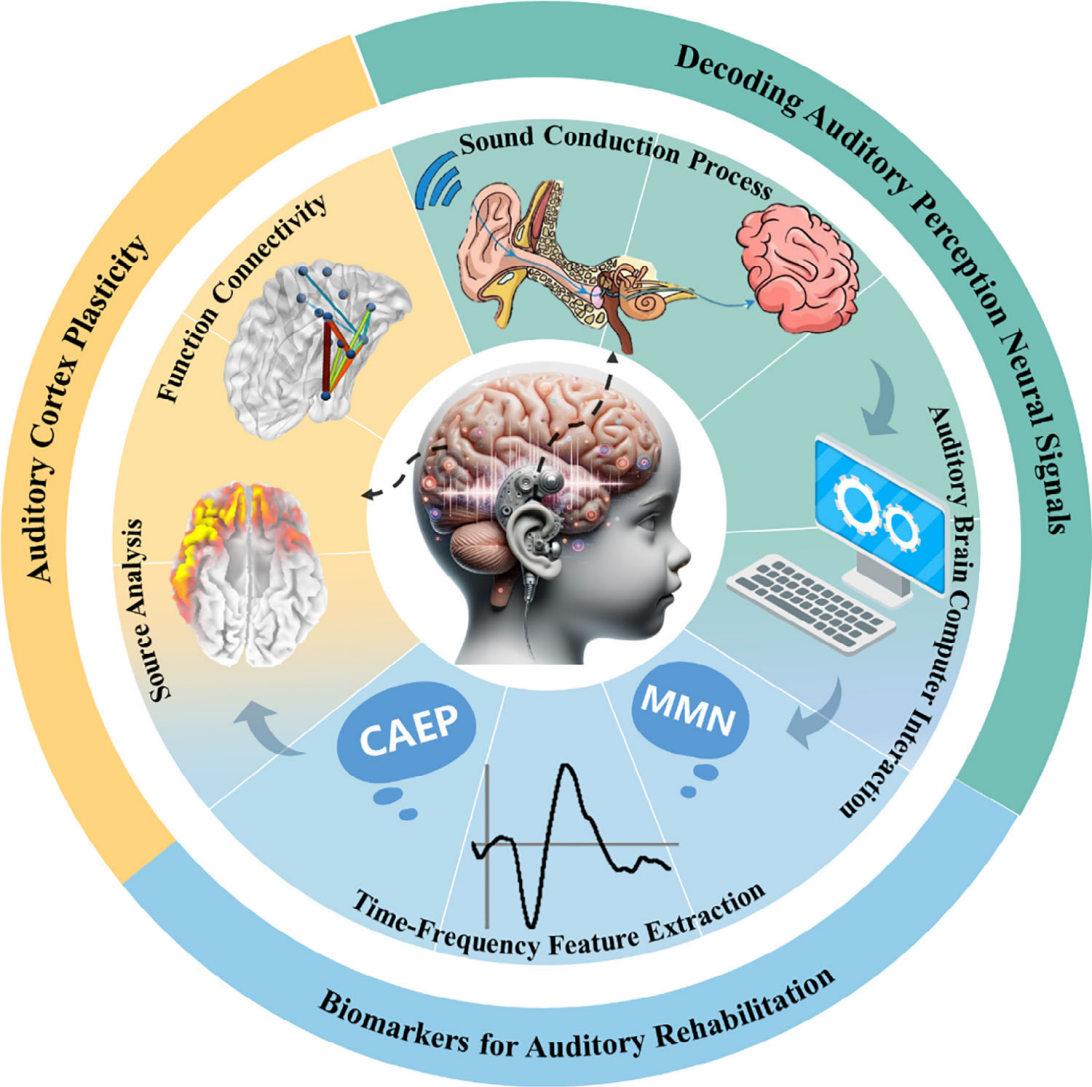
The application of ABCI technology in assessing auditory abilities in children with CIs is summarized. ABCI connects the brain with external devices and decodes neural signals related to auditory perception. Time‐domain and frequency‐domain analyses are commonly used to identify biomarkers associated with auditory rehabilitation, such as cortical auditory evoked potentials (CAEP) and mismatch negativity (MMN). Reproduced with permission [43]. Copyright 2006, Elsevier. Additionally, source and functional connectivity analyses are employed to elucidate the process of auditory function reconstruction post‐implantation from the perspective of cortical neural plasticity. Reproduced with permission [46]. Copyright 2021, Elsevier.

This review offers an overview of the application of ABCI in objectively evaluating the advanced auditory functions of CI users (Figure 1). It reviews ABCI assessments of various auditory functions along a hierarchical continuum, including the perception of sound's physical properties, non‐linguistic music perception, and language perception. Additionally, it introduces a detailed study of cortical neural plasticity following CI implantation, exploring the process of auditory function reconstruction in depth. This review aims to highlight unresolved challenges and provide direction for future research to advance the application of ABCI in auditory assessments for CI users.

## 2 ABCI in Assessing Auditory Function

**FIGURE 2:**
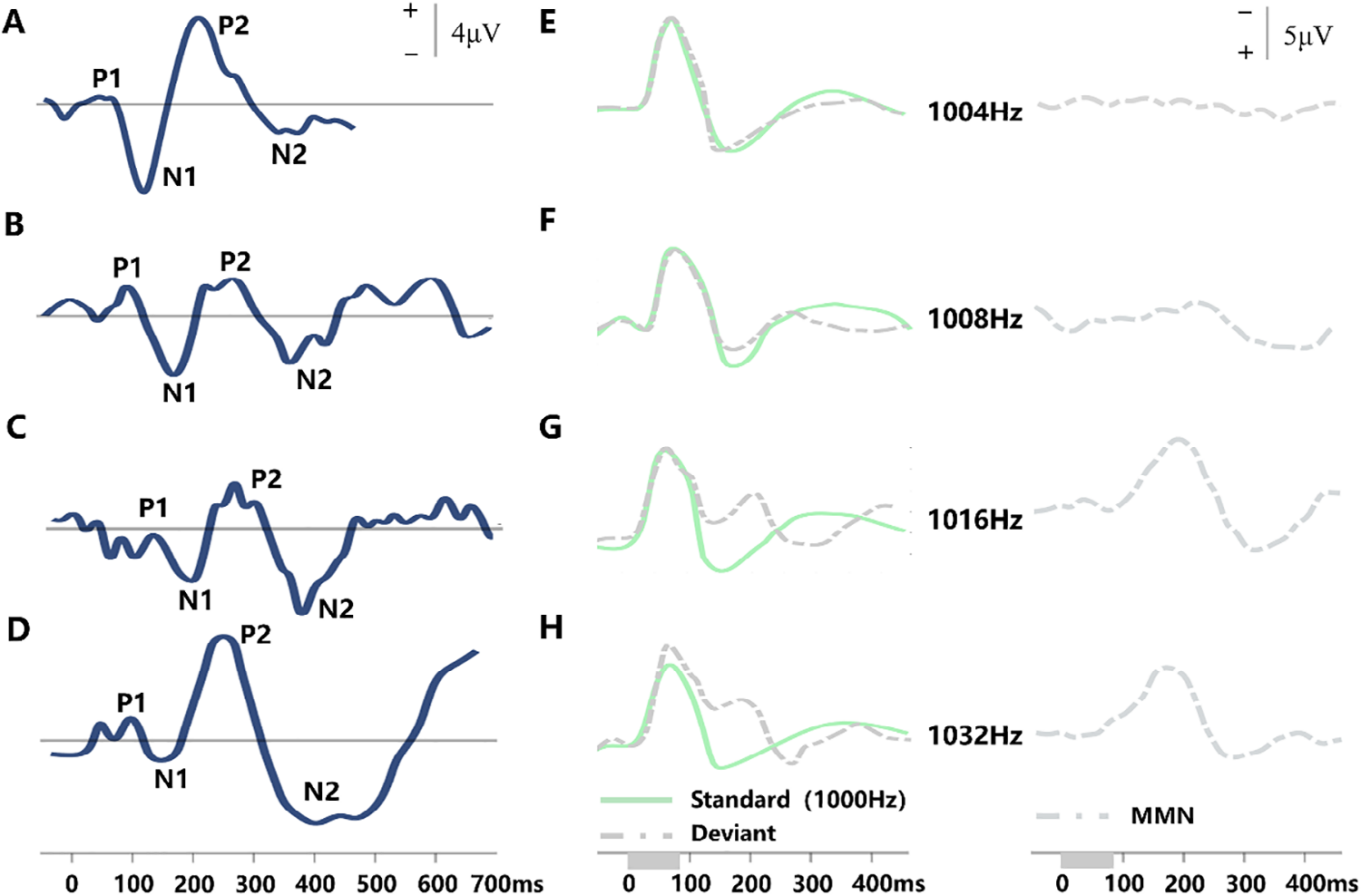
(A) CAEP responding to the word “bad’’ at Cz in adults (*n* = 9), (B) 4–6 years old (*n* = 20), (C) 1–3 years old (*n* = 19), (D) newborns (*n* = 8) from top to bottom. Newborns typically lack P1 and N1. The auditory perception ability is determined by observing the latency and amplitude of the P1 wave. Reproduced with permission [43]. Copyright 2006, Elsevier. (E) The left side is ERP to 1000 Hz standard and deviant stimuli of 1004 Hz, (F) 1008 Hz, (G) 1016 H and (H) 1032 Hz. The right side is the MMN obtained by subtracting the ERP caused by the standard stimulus from those caused by different deviant stimuli. The auditory recognition ability is examined by observing the absence, latency, and amplitude of the MMN. Reproduced with permission [31]. Copyright 1978, Elsevier.

The brain's electric potential fluctuations, which occur during the presence or absence of sound, precisely and consistently represent the processing of auditory stimuli responses [39, 40]. The brain generates numerous event‐related potentials (ERPs) related explicitly to auditory processing during perception, recognition, and sound memorization [41]. Some components of these ERPs, such as CAEP (P1, N1, P2, N2), MMN, P300, etc., serve as biomarkers in the auditory assessment of CI recipients. The P1 component [42], an early event‐related potential, typically appears around the 100‐ms post‐stimulus presentation. This component symbolizes primary perceptual processing, especially early sound perception, and basic sound properties, like intensity and frequency. Moreover, the latency of P1 gradually shortens with age until adulthood [43], as shown in Figure 2A–D. N1 [42], another early ERP, manifests 150 to 200 ms following a stimulus presentation and concerns sound recognition and attention allocation. It demonstrates greater amplitudes when paying attention to a sound. Näätänen first proposed and verified MMN as a negative ERP component (Figure 2E–H) [31], observable between 150 and 250 ms after stimulus presentation. The induction of MMN happens when an unusual stimulus is interspersed with frequent standard stimuli. This unusualness can reflect differences in stimulus intensity, frequency, or duration [33], implying the brain's sensitivity to unanticipated or discordant sound events. Furthermore, MMN is pivotal in research involving auditory discrimination and memory. P2 [42], a positive potential emerging between 200 to 250 ms following stimulus presentation, indicates superior sound processing aspects—for instance, sound recognition and language processing. N2 [44], a negative potential that surfaces approximately 250 to 350 ms post‐stimulus, is associated with speech recognition and syntactic processing. N2 typically surges when facing grammatical errors or regulatory non‐compliant sounds. The P300 [36], a late ERP, commonly manifests within 300 to 500 ms after a stimulus. Furthermore, certain ERP components exhibit sensitivity to language‐related factors. Notably, the N400 [45], a prominent language‐related component, is characterized by a negative waveform with its zenith predominantly seen at the central and vertex electrode locations. It pertains to attention, memory, and decision‐making processes and is a popular tool for studying cognitive mechanisms in intricate auditory tasks.

Identifying biomarkers from brain activity is vital and complex, specifically for the auditory rehabilitation of children fitted with CIs. These biomarkers provide an objective gauge of auditory function and play a crucial role in evaluating the effectiveness of the implant in enhancing auditory capabilities. They can be employed to monitor the progression of auditory discrimination, music recognition, and speech comprehension among CI users, thus assessing the ongoing impact of the CI experience on auditory skills. Importantly, these biomarkers can accurately complement behavioral assessment results in restrictive behavioral tests, such as tests for pediatric CI patients. This section summarizes the perceptual evaluations of sound physical properties, nonverbal music, and language at three levels in the objective evaluation of CI users' auditory function using ABCI. By summarizing how various components of auditory brain activity, as reflected in ERPs, serve as biomarkers, this section aims to illustrate the importance of these biomarkers in objectively evaluating CI users' auditory abilities.

### 2.1 Perception of Acoustic Physical Properties

The P1, N1, and P2 components of CAEP often reflect cortical activity within the cortical‐thalamic circuit [47, 48]. These components can vary due to alterations in sound properties and differ among individuals and ages, thereby providing insights into auditory perceptual capacities. Recent focus has been on developing P1 cortical auditory evoked potentials in patients with sensorineural hearing loss post‐cochlear implantation. Kátia de Freitas Alvarenga and her team conducted a longitudinal study involving ten prelingual sensorineural hearing loss children who underwent cochlear implantation [49]. The CAEP study utilized /da/ speech stimuli delivered in a free field at three distinct points: activation of the CI, three months post‐activation, and six months post‐activation. Results pointed to a reduction in the latency and amplitude of the P1 component with increased use of the CI. The shift in the latency of the P1 component in CAEP found that six months after CI activation, the central auditory system's maturity in children implanted within the auditory sensitive period could approximate normal levels [50], and the rate of maturity is similar to that seen in children with normal hearing, although delayed by the corresponding auditory deprivation time [51].

Other researchers corroborated these findings [52], noting a significant decrease in the latency period of the P1 component three months after implantation upon analyzing changes in the morphology and latency of P1‐N1‐P2 components in both pre and post‐cochlear implant surgery. The younger the recipient at the time of CI surgery, the larger the decrease in the latency period of the P1 component, suggesting that auditory pathways can mature rapidly after electrical stimulation [53]. Sharma and colleagues reviewed the research evidence of P1 and N1 cortical auditory evoked potentials in children with congenital deafness and children with auditory nerve spectrum disorders who underwent cochlear implantation at varying ages during childhood, supporting the clinical utility of P1 in CAEP as a non‐invasive biomarker. Furthermore, among patients with sensorineural hearing loss and auditory nerve disease, P1‐N1‐P2 components are only present in children demonstrating robust speech perception abilities [54].

A fascinating study contrasted the cortical processing of pitch information in acoustic and electric hearing in subjects with unilateral deafness, where one ear had a CI, and the other ear exhibited normal hearing. The results indicated that auditory cortical activity in electric hearing was reduced and delayed, especially regarding the latent components associated with the P2 event, pointing to discrepancies in the processing of pitch information between the two hearing types [55]. A study by Darren and colleagues found that a single‐channel CAEP could be used to estimate the threshold level of CI users [56]. They created a fully objective method to estimate the threshold level of CI users through the growth function fitting and the peak‐locked value characteristics, which demonstrated a strong correlation with behavioral thresholds. Additionally, through analyzing various CAEP components, audiologists can determine if the CI provides appropriate stimulation and utilize it to adjust auditory rehabilitation plans. When a component is absent in CAEP, the device is adjusted until all components in CAEP are significantly induced, thereby optimizing the comfort of CI users and swiftly and objectively verifying the applicability of CI [57]. Although the study only sampled adult CI users, it is anticipated that if CAEP is used as an evaluation tool in the initial stages after children's implantation, it would also benefit CI users during the hearing rehabilitation process.

MMN offers a means to measure the brain's ability to automatically identify changes in sound, irrespective of subjective selectivity and attention [58]. MMN has been effective in assessing the auditory recognition capabilities of populations. It has been identified that the latency and amplitude of the MMN peak are connected to the speech recognition effects of adult CI users [59, 60]. CI users with varying speech recognition capabilities have different MMN morphology. CI users with good language recognition have larger amplitude MMN [32], similar to those witnessed in individuals with normal hearing ability, while those with average to low language scores indicate smaller amplitudes or even absent MMN [61]. A longitudinal comparison found that a reduction in the latency period of the MMN within the 3 to 6 months post‐CI implantation was significantly tied to the subjective hearing ability development score [62].

Contrary to distinguishing different frequency pure tones, children with normal hearing appear to have higher amplitude and longer latency MMN when distinguishing pitch, but CI children do not manifest this effect. Compared to peers with normal hearing, CI children frequently present with longer latency periods and lower amplitudes [63], suggesting that the auditory system of CI children is not yet fully developed. Although a distinction could be identified between non‐language pairs (i.e., 1000 and 1500 Hz pure tones) and language pairs (i.e., /ba/ and /ka/), the auditory system still exhibits a time delay [60].

In another study, Ni and others tested 91 children aged between 3–7 years, including 66 CI children and 25 children with normal hearing [46], to identify a universal change pattern post‐cochlear implantation in CI children. The study utilized frequency, syllables, and tonal sounds to evaluate the auditory rehabilitation status post‐implantation. It was reported that there is a critical stage for rapid development for simple language reconstruction between 3 to 6 months post‐implantation, and 6 to 12 months was pivotal for language tone recognition. This suggests that CAEP is suitable for evaluating early auditory system reconstruction, and MMN may serve as a biomarker of CI children's sound discrimination abilities post‐implantation.

The automatic and non‐attention‐necessitating features of CAEP and MMN make them formidable tools for studying auditory processing, diagnosing auditory‐related disorders, and exploring the auditory functionalities of distinct populations. They furnish information independent of subjective consciousness, facilitate the detection of auditory abnormalities and aid in assessing auditory perception and recognition functionalities. As a neurophysiological tool, it displays a significant utilization potential in auditory function assessment.

### 2.2 Non‐Linguistic Music Perception

Music perception is a complex task that the human auditory system can perform, demonstrating the integration of the auditory system with brain functions [64]. Music shares synergies and origins with language, and it can bolster language abilities and cognitive functions for children [65]. Fundamental musical elements such as pitch, timbre, and rhythm characterize musical features. Researchers heavily utilize these elements for music perception evaluation. Studies using EEG technology for music perception typically deconstruct music into various features, allowing categorization of specific musical traits and facilitating quantitative analysis of music‐related brain electrical particulars. Currently, research on music perception in CI children mainly revolves around the recognition capability of these fundamental music elements and a holistic understanding and perception of music. Distortions in timbre and pitch that trigger MMN and P3a display extended latency and decreased amplitudes in the brain response of CI children compared to their normal counterparts [66]. This evidences CI children's lesser proficiency in identifying pitch and timbre.

**FIGURE 3:**
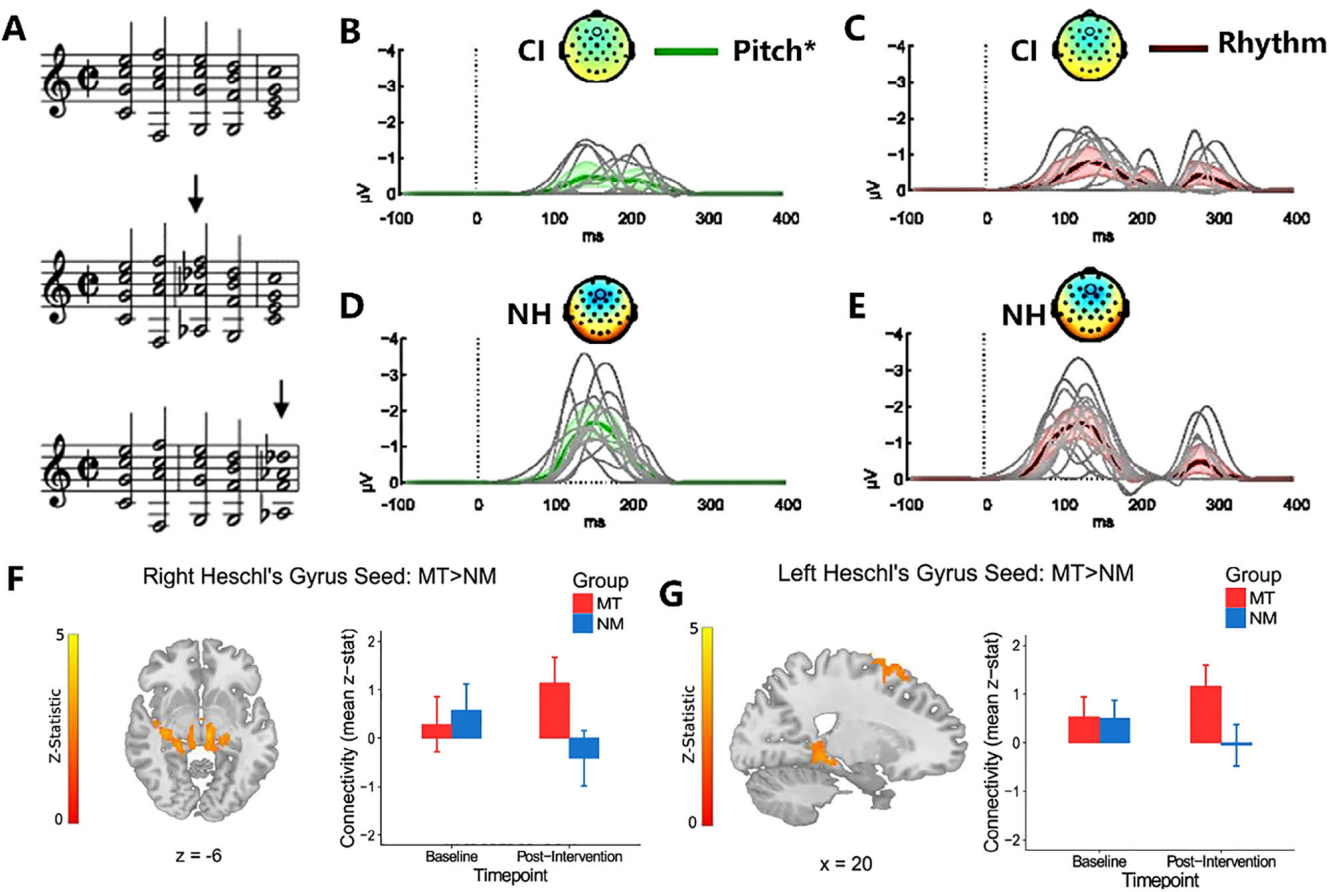
(A) A schematic diagram of music stimulation. Reproduced with permission [67]. Copyright 2024, Elsevier. (B) The performance of the CI group and (C) normal hearing (NH) group under pitch stimulation, and the (D) CI group and (E) NH group under rhythmic stimulation. It is grand‐average difference waveforms with spike density component analysis (SCA) statistics. Difference waveforms are shown for the CI users and NH groups for each deviant feature and magnitude. Reproduced under the terms of the Creative Commons CC‐BY license [68]. Copyright 2023, The Authors. (F) It shows regions of increased resting‐state functional connectivity (RSFC) post‐intervention in the Music (MT) vs. Non‐music (NM) groups between Right Heschl's gyrus seed and subcortical regions such as the hippocampus and thalamus and (G) left Heschl's gyrus seed and fronto‐motor regions. Reproduced under the terms of the Creative Commons CC‐BY license [71]. Copyright 2018, The Authors.

In 2004, Koelsch et al. investigated the coherence judgment of musical phrases between CI users and normal‐hearing populations (Figure 3A) [67]. Both groups generated MMN to harmonic abnormalities in music sequences. Observed MMN and P3 components verified CI users' perception of harmonic anomalies. Additional research explored the neurophysiological mechanisms behind music pitch recognition of Mandarin‐speaking adolescent CI users and NH listeners (Figure 3B–E) [68]. This disclosed an inferior music pitch discrimination ability in CI users, reflected in increased latency and decreased mismatch negativity amplitude, indicating MMN as a potential indicator of cortical pitch perception in Mandarin‐speaking CI users [69]. ERP responses also disclosed weaker timbre perception differences in CI hearing. Rahne et al. determined that MMN components can objectively measure the ability of CI users to distinguish timbre [70]. CI users only induced MMN for timbre changes above their subjective minimum discernable threshold, significantly weaker than the normal hearing control group. MMN responses revealed the incapability of CI hearing to discern timbre differences caused by differing temporal envelopes and spectral distribution. The discussed studies suggest that MMN can be an effective method to evaluate the ability of hearing‐impaired patients to perceive music.

Other studies implicitly stated that musical activities or music training can influence auditory perception ability [72]. Pantev et al. reported that short‐term musical activity or training intervention could also affect hearing ability [73, 74], as also found by Sharda et al. [71], as shown in Figure 3F,G among children. After 15 months of music training, the auditory perception abilities of children aged 5–6 improved [75], evident as structural changes in the brain's temporal, frontal, and parieto‐occipital lobes. These changes correlate with those found in adult musicians. Fu et al. explored the melody recognition of CI children and the effects of short‐term music training using the melodic contour identification (MCI) task to evaluate the ability of 5–10‐year‐old Mandarin‐speaking CI children to recognize and understand melody [76]. MCI melodies are typically short, and this task requires participants to recall the first melody and identify changes in the target melody [77]. The CI children achieved an average accuracy rate of only 33.3% in the MCI instrument timbre and melody note length recognition task. After four weeks of home‐based music training, the MCI performance significantly improved and did not decrease eight weeks after the completion of the test.

Cheng et al. also used MCI to investigate the impact of music training on music melody recognition, speech intonation, and sentence perception of CI children [78]. They found that after eight weeks of music training, Mandarin‐speaking CI children aged 5–10 improved their perception abilities of melody, tone, and sentence, and their performance did not change significantly even four weeks after the training stopped. However, Peterson et al. documented significant differences in pitch, timbre, intensity, and rhythm perception between CI children and normal children after two weeks of music training [26]. The neurophysiological responses and behavioral results concurred, but noticeable differences before and after music training were absent, possibly due to the simplicity and insufficient duration of the music training program. Kosaner et al. enacted an 18‐month music training and rehabilitation plan, which enabled 25 CI children to identify pitch, timbre, and rhythm [79]. Systematic music training enhances music perception ability and significantly enriches their music experiences. It is posited that systematic and comprehensive music activities and training should be provided to pre‐lingually deaf CI children before school age to assist in developing auditory perception skills. In similar research, Torppa et al. identified changes in the generation of P3 and MMN responses, and singing had a more marked influence on the P3 responses of CI children [66]. This attests to the idea that music training not only improves the music perception abilities of the hearing‐impaired but can also objectively and effectively evaluate this phenomenon through neurophysiological indicators. These findings yield valuable insights into the intricate relationship between music and brain activity and underscore the potential of electroencephalogram in understanding music perception and as an assessment tool.

### 2.3 Language Comprehension

**FIGURE 4:**
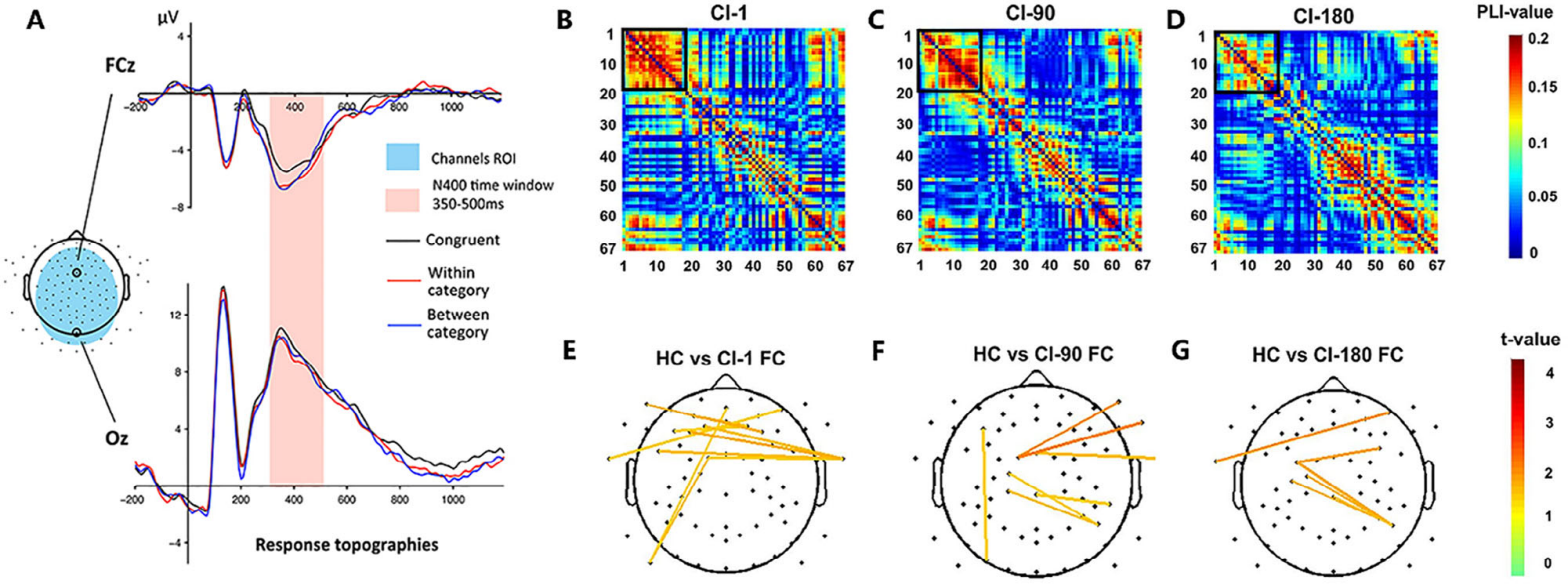
(A) Overview of N400 responses: Average responses at FCz and Oz. Averages include all participants, collapsed across pre‐ and post‐intervention. Reproduced under the terms of the Creative Commons Attribution License (CC BY) [83]. Copyright 2016, The Authors. (B) The standard stimulus function connection analysis in the follow‐up study. Reproduced under the terms of the Creative Commons Attribution License (CC BY) [85]. Copyright 2021, The Authors. The values of the standard stimulus phase lag index (PLI) for 67 electrode pairs in the group implanted with CI for 1 day, (C) 90 days, (D) 180 days. (E) The *t* scores adjusted for *p* < 0.005 and differential functional connection across the topography between NH group and CI‐1 group, (F) CI‐90 group, (G) CI‐180 group.

The main emphasis of prior studies lies prominently in the understanding and reception of various auditory elements. Yet, language comprehension, comprising vocabulary and syntax, presents a deeper perspective garnering academic attention. Using ERP as an evaluative tactic poses no age‐related constraints and mirrors the semantic recognition process. Established research evidences the ability of infants as young as six months to set up semantic associations between words and objects provisionally. This effect, however, is transient and disappears within a day. It takes until their second year for a sustainable semantic relationship to be established [80]. Advancing age sees children with CIs grapple with understanding spoken language, attributed to defects in low‐level acoustic encoding and elevated cognitive processing [81]. An EEG study revealed that children with CIs demonstrate decreased mismatch negative waveforms and lack late discriminative negativity (LDN) compared to their normal‐hearing peers. This signifies impairments both in auditory sensory and cognitive processing [82]. Contemporary research explores the response of the N400 component to semantic processing. Initially observed and defined in a six‐year‐old, researchers expanded their study sample to include children with CIs and hearing aids alongside those with normal hearing. Using an audio‐visual combined stimulant approach, they investigated the semantic understanding capabilities of children aged between five and seven. The study revealed N400 presence across all groups, as shown in Figure 4A, but stopped short of testing statistical disparities [83]. Expanding on this, they experimented using a matching and non‐matching word‐picture stimulus on 38 pre‐lingually deaf children with CIs implanted between nine and fifty months. EEG readings recorded after 12, 18, and 24 months of implantation indicated that those who scored below the standard range in language tests displayed negligible N400 effect. In contrast, those meeting or exceeding the standard revealed an incrementally apparent N400 [84]. Thus, inferring N400 could be a semantic comprehension biomarker is plausible.

Conducting a longitudinal study, EEG signals from ten CI users were recorded when the implant was activated and again at 180 days post‐activation. The purpose was to evaluate the neural processing of speech in CI users by studying the oscillatory features of their neural response to asynchronous syllables and cross‐frequency coupling at multiple frequencies. The study proposed that phase‐amplitude coupling could be a biomarker for assessing speech function recovery in CI users [86]. Additional research incorporated EEG and behavioral indices reflecting auditory function remodeling in CI patients at multiple time intervals post‐implantation (1, 90, and 180 days). Using the Chinese Word and Sentence Lists to gauge speech perception abilities and analytical methods such as phase lag index (PLI) and functional connectivity, results indicated an improvement in speech recognition capabilities over an extended duration of the implant. Even though implant patients identified stimulus differences similar to the control group, there were significant variations in latency periods and functional connectivity between the two groups. N1 amplitude in the alpha waveband, N1/P2/MMN delay, and whole‐brain functional connectivity based on PLI were seen as potential indicators reflecting the remodeling process of auditory functions. These parameters serve as potential objective measures for evaluating speech perception abilities and the impact of cochlear implantation [85], as shown in Figure 4B–G.

Overall, EEG's immediacy and accuracy in providing feedback on response toward varying types of auditory stimuli prove invaluable, especially for children with CIs who may not respond through speech or behavior. Thus, the efficacy of CIs and a child's auditory and linguistic understanding capabilities gain elucidation through biomarkers, as shown in Table 1. Moreover, the objectivity of EEG data ushers the possibility of personalized CI adjusting.

**TABLE 1:** Potential biomarkers of ABCI in auditory assessment.

Biomarker	Explanation	Reference
P1	Biomarker for primary perceptual processing, early sound perception, and basic sound properties like intensity and frequency.	[46, 49, 50, 51, 52, 53, 54]
CAEP	Directly used to estimate the threshold level of CI users and assist in adjusting CI processor.	[55, 56, 57]
MMN	Biomarker for automatically recognizing sound changes, including differences in stimulus intensity, frequency, duration, pitch, timbre, rhythm, and so forth.	[32, 46, 59, 60, 61, 62, 63, 69, 70]
P300	Biomarker for evaluating pitch, timbre, and rhythm perception under attentional state.	[66, 67, 68]
LDN	Biomarker in auditory perception and cognitive processing.	[82]
N400	Biomarker for semantic understanding.	[83, 84]
Phase‐amplitude coupling	Potential biomarker for assessing speech function recovery, evaluating the neural processing of speech by studying the oscillatory features of neural responses.	[86]
PLI	Potential indicators of the auditory function remodeling process.	[85]

### 2.4 Comparative Clinical Utility of Traditional Methods and ABCI‐Based Biomarkers

Having discussed the application of ABCI in evaluating auditory function, it is essential to compare the practicality of these promising biomarkers with traditional auditory assessment methods to understand their respective advantages and limitations.

Traditional methods, such as pure‐tone audiometry and speech recognition tests [23], have been widely used for their straightforward implementation and direct interpretation of results. However, these methods rely heavily on behavioral responses [87], which can be challenging to obtain from pediatric patients or those with severe communication difficulties.

In contrast, biomarkers derived from ABCI technology, such as CAEP and MMN [31], offer objective measures of auditory function that do not depend on the patient's active participation. These neurophysiological markers provide continuous and precise monitoring of auditory processing, enabling clinicians to assess the effectiveness of cochlear implants more accurately and tailor rehabilitation programs accordingly.

Moreover, using ABCI technology allows for the detection of subtle neural changes that may not be apparent through traditional methods. For example, CAEP components can indicate early perceptual processing and neural plasticity, while MMN can reflect automatic auditory discrimination abilities [46]. These insights are invaluable for developing personalized intervention strategies and tracking long‐term auditory development in cochlear implant users. Table 2 compares traditional auditory assessment methods and ABCI‐based biomarkers to illustrate these points further.

**TABLE 2:** Comparison of traditional methods and ABCI‐based biomarkers.

Criteria	Traditional methods	ABCI‐based biomarkers
Patient participation	Requires active participation	Does not require active participation
Ease of implementation	Straightforward, widely used	Requires specialized equipment and expertise
Measurement type	Behavioral	Neurophysiological
Assessment scope	Limited to behavioral responses	Continuous monitoring of neural changes
Sensitivity	May miss subtle neural changes	Detects subtle neural changes
Applicability	Suitable for most patients	Useful for pediatric or non‐verbal patients
Data interpretation	Direct and intuitive	Requires specialized knowledge
Long‐term monitoring	Less effective	Effective for long‐term monitoring
Tailoring interventions	Limited by behavioral data	Enables personalized strategies

By integrating these advanced biomarkers with conventional auditory assessments, clinicians can achieve a more comprehensive evaluation of auditory function, leading to improved outcomes in auditory rehabilitation. Thus, combining traditional methods and ABCI‐based biomarkers holds significant potential for enhancing the clinical management of cochlear implant patients.

## 3 ABCI in Exploring Cortical Neuroplasticity After Cochlear Implantation

In addition to evaluating auditory function, exploring how ABCI technology can be utilized to understand cortical neuroplasticity following cochlear implantation is crucial. While the application of ABCI in assessing auditory function provides critical insights into the effectiveness of CIs in improving hearing capabilities in pediatric patients, examining cortical neuroplasticity offers a deeper understanding of the brain's adaptive changes post‐implantation. This transition from functional assessment to exploring neural plasticity enables a more comprehensive view of the impact and potential of ABCI technology in immediate and long‐term auditory rehabilitation for children with CI.

After cochlear implantation, the auditory cortex commences the reception and processing of auditory information from the implant. This initiates a neural remodeling in the auditory cortex, a process that requires time and is not yet fully understood. During periods of hearing loss, the auditory cortex may experience invasion by other sensory systems [88], including but not limited to vision and touch. Upon reestablishing auditory function through cochlear implantation, the auditory system may reoccupy the previously invaded territories [89]. Furthermore, prolonged use of the CI can induce structural and functional adaptations in the cerebral regions responsible for speech and language processing, accommodating auditory inputs from the implant [90]. Electroencephalographic studies indicate changes in neural activity patterns and activation of discrete cerebral regions during auditory tasks with Cis [73].

To investigate these phenomena, we reviewed the use of ABCI technology to explore cortical neural plasticity following cochlear implantation. First, we examined the changes and adaptations in the auditory cortex post‐implantation, discussing how ABCI can track these neural changes and evaluate their impact on auditory processing. Next, we explored the phenomenon of cross‐modal plasticity, where non‐auditory sensory modalities interact with and affect the auditory cortex. This section emphasizes how ABCI technology can provide a deeper understanding of the complex neural interaction mechanisms involved in auditory rehabilitation.

### 3.1 Auditory Cortex Remodeling

**FIGURE 5:**
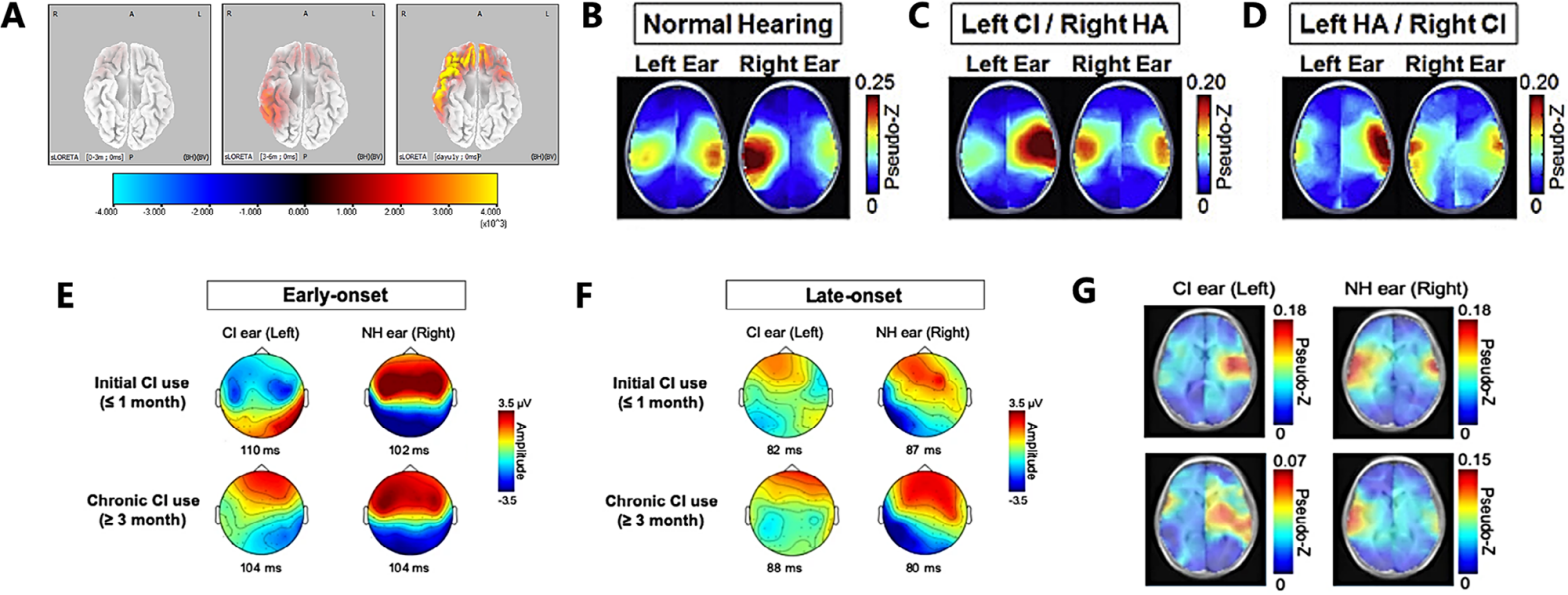
(A) Cortical activation in children with different CI durations, separately in 0–3 months, 3–6 months, 12 months, and above. Reproduced with permission [46]. Copyright 2021, Elsevier. (B) Cortical organization in children with asymmetric hearing. The activated regions during the P1 period when stimulating the left and right ears show that activity across the NH group, (C) left CI/right HA, (D) left HA/right CI, are all confined to the auditory cortex. Reproduced with permission [94]. Copyright 2018, Elsevier. (E) Cortical effects of single‐sided deafness in children at early‐onset and (F) late‐onset of initial CI use. Topographic distribution of mean average‐referenced EEG activity across the surface of the head at the peak latency. Reproduced under the terms of the Creative Commons Attribution License (CC BY) [95]. Copyright 2020, The Authors. (G) Axial view of mean evoked source activity that shows the highest activation in the auditory cortex contralateral to the stimulated ear. Reproduced under the terms of the Creative Commons Attribution License (CC BY) [95]. Copyright 2020, The Authors.

The cortical remodeling process post‐cochlear implantation, especially in pediatric recipients, is a focal point of current research. By comparing the brain responses to auditory stimuli pre and post‐cochlear implant usage, researchers are evaluating this transformation within the auditory cortex [91]. Studies suggest that sound source localization effectively investigates the plasticity of the auditory cortex, thereby providing valuable insights for evaluating auditory rehabilitation programs. Observations indicate a minor activation in the right temporal lobe from post‐implant device activation up to 3 months, with increased activation observed after 6 months. Upon a year or more of CI usage, the activation region was found to spread to the left frontal and temporal lobes, with increased activation intensity. However, statistical analyses revealed persistent disparities between children with CIs at all three stages of implantation and their normal counterparts (Figure 5A) [46]. Although cochlear implantation encourages swift development of the auditory cortex in the early stages, achieving normative hearing levels in a short period is unfeasible due to insufficient auditory experience [92]. Additional investigations have explored whether late cochlear implantations could foster cortical development beyond the optimal developmental period. Findings show that auditory components of a hearing‐impaired ear surface only six to eight months post‐implantation in children of mean age 9.86 years, affirming a substantial level of neural plasticity in the auditory cortex despite late implantations [93]. In addition, by quantifying the functional connectivity between channel pairs and the synchronization of brain network activity within regions of interest and utilizing these synchrony values as features for SVM classifiers, EEG‐based brain network synchronization analysis was able to differentiate children with CI from pre‐lingually deaf children without CI. This underlines the restructuring of brain functional networks post‐cochlear implantation.

Furthermore, research finds that the normal development of cortical networks for sound processing requires bilateral input in children with a CI [96]. In contrast, asymmetrical cerebral cortex development might lead to language impairments. Nevertheless, an asymmetrical evolution of the cerebral cortex can give rise to language deficits [97]. Notably, unilateral auditory deprivation in infancy can induce cortical augmentation on the affected side [98]. However, even after restoring bilateral input in subsequent years, the cortical disequilibrium remains, indicating challenges in mitigating the impacts of prolonged unilateral hearing loss [99]. To compensate for the un‐implanted ear, children commonly wear hearing aids. Studies have investigated the cortical responses to bimodal stimuli in children with asymmetric hearing who receive a CI in one ear and use a hearing aid in the other. Research finds that early provision of balanced bimodal stimulation can prevent the development of cortical bias and effectively improve speech perception in these children (Figure 5B–D) [94]. Studies have also compared brain activity before and after receiving the second implant to better understand the impact of bimodal integration on users. The participants included ten children who experienced an average period of 3.1 years of hearing asymmetry before implantation. The results suggest that the new implant stimulates the activity in the ipsilateral auditory cortex. However, the resolution is limited in children with a longer duration of hearing asymmetry, implying that early implantation of the second implant can restore the manifestation of binaural auditory input in the auditory cortex. Moreover, the continuous long‐term use of CIs can partially reverse the cortical effects of single‐sided deafness (SSD) in children, indicating a positive impact of CI use on cortical reorganization (Figure 5E–G) [95].

### 3.2 Cross‐Modal Reorganization

**FIGURE 6:**
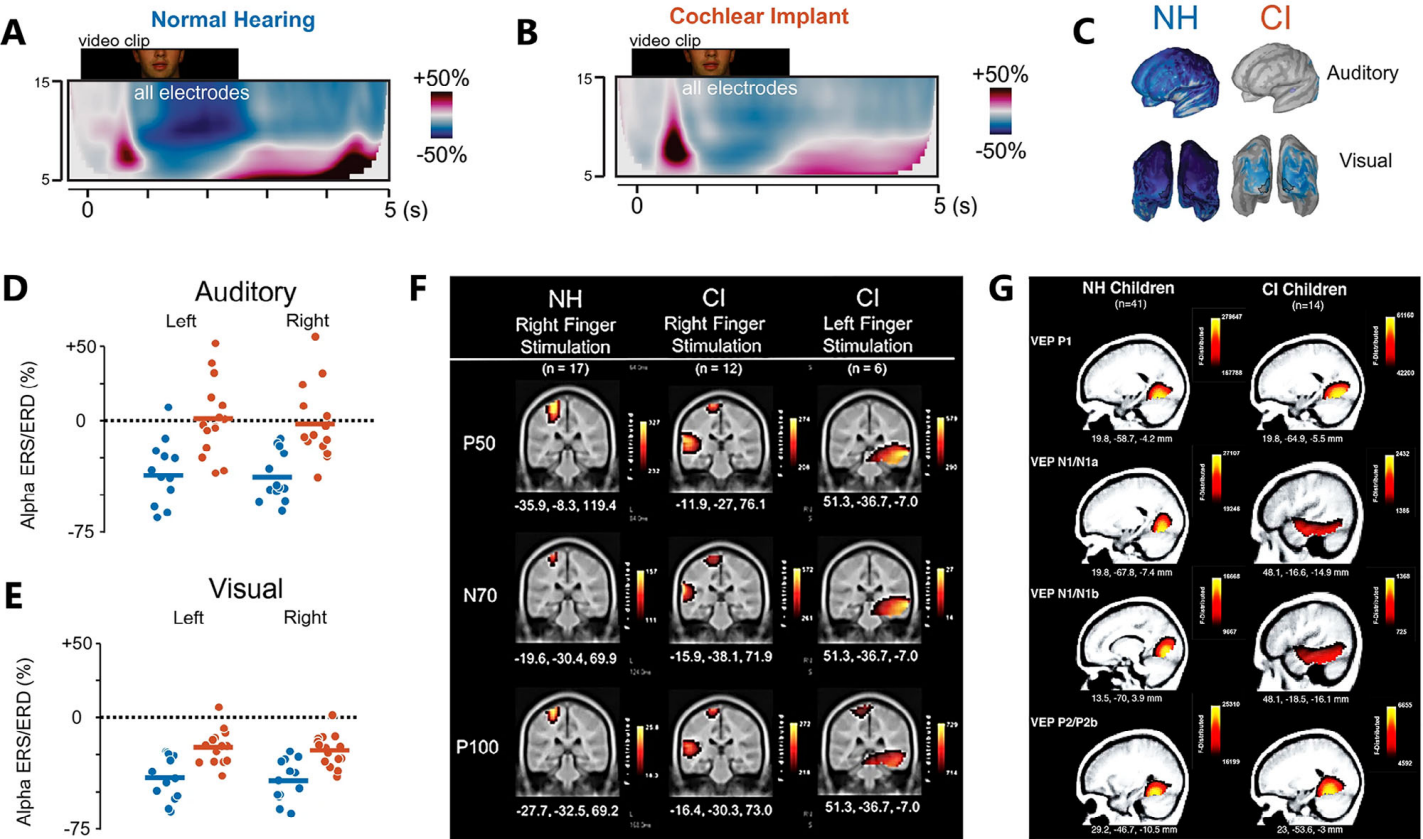
(A) EEG event‐related synchronization and desynchronization (ERS/D) across frequencies expressed as a percent change of power from baseline for all electrodes in NH and (B) CI group under video clip. Reproduced under the terms of the Creative Commons CC BY license [35]. Copyright 2022, The Authors. (C) Whole‐brain plots show alpha power ERS/ERD distributions from left frontal and posterior views between CI and NH groups. Reproduced under the terms of the Creative Commons CC BY license [35]. Copyright 2022, The Authors. (D) Activity in auditory and (E) visual ROIs in left and right hemispheres for the source of the ERD is shown for individual subjects in CI (red) and NH (blue) groups. Reproduced under the terms of the Creative Commons CC BY license [35]. Copyright 2022, The Authors. (F) Somatosensory cross‐modal reorganization. CDR for cortical somatosensory evoked potentials (CSEP) in normal hearing and cochlear‐implanted children. Cortical activations in response to vibrotactile stimulation of the right index finger in children with NH and CI. Activations are organized in rows corresponding to each CSEP waveform component (P50, N70, P100). CDRs are presented on coronal slices for each of these components. Reproduced under the terms of the Creative Commons Attribution License (CC BY) [103]. Copyright 2016, The Authors. (G) Visual cross‐modal reorganization. Current density reconstructions (CDR) for visual evoked potentials (VEP) in NH and CI children. Cortical activations for each VEP peak component (P1, N1, P2) are shown for NH and CI groups. Reproduced under the terms of the Creative Commons Attribution License (CC BY) [104]. Copyright 2019, The Authors.

Cross‐modal reorganization refers to repurposing unused cortical areas by other sensory systems when sensor inputs are absent. In recent years, the cortical cross‐modal reorganization in CI children has been an emerging hotspot. Most related studies focus on the cross‐modal reorganization between vision and hearing when investigating the cross‐modal cortical reorganization of CI users and their relationship with audio‐visual integration skills (Figure 6A–E) [35]. These studies have found that compared to normal hearing, CI users show stronger cross‐modal activation in their auditory cortices [100]. Furthermore, a positive correlation exists between CI users' cross‐modal activation and audio‐visual integration strength [101]. Additionally, CI users demonstrate plasticity in the visual cortex, increasing the recruitment of the visual cortex for motion‐processing tasks and indicating compensatory changes in the visual cortex due to hearing loss [102]. Differences in the source estimation of P1, P2, and visual MMN components between CI users and NH participants highlight the complex interplay between deafness and brain plasticity (Figure 6F) [103]. An examination of somatosensory cross‐modal reorganizations in CI children using high‐density EEG revealed the activation of the auditory cortices during somatosensory stimulation, suggesting cross‐modal reorganization.

Interestingly, the degree of cross‐modal reorganization was related to speech perception results (Figure 6G) [104]. Moreover, compared to normal hearing children and early implanted cochlear children, pre‐lingually deaf children receiving late cochlear implantation exhibit global network deficits in brain networks. This suggests that auditory deprivation has long‐term effects on brain networks, but early implantation within a sensitive period can help cochlear‐implanted children recover brain network characteristics. Most importantly, the correlation between brain network characteristics and speech perception scores suggests that brain network indices could reflect the degree of recovery after cochlear implantation [105].

Research on the cross‐modal reorganization in cochlear‐implanted children is currently limited, but it provides valuable insight into how the brain processes and decodes sound, especially language, after implantation. This information deepens our understanding of how the brain works after implantation and provides an important reference for optimizing post‐implant assessment and intervention techniques.

### 3.3 Comparative Analysis of EEG and Other Neuroimaging Techniques

Various neuroimaging techniques, such as fMRI, PET, MEG, and near‐infrared spectroscopy (NIRS) [91, 106, 107], can be used to study auditory cortex activity. Among these, EEG is widely used due to its high temporal resolution, allowing for real‐time neural activity monitoring. This is particularly useful for evaluating rapid changes in auditory processing and neural plasticity after cochlear implantation. Additionally, EEG is relatively non‐invasive and cost‐effective, making it suitable for regular clinical use. However, its spatial resolution is limited. Although high‐density EEG can somewhat compensate for this limitation [108], it still cannot accurately locate deep brain activity.

In contrast, fMRI offers excellent spatial resolution, enabling precise localization of brain activity. This makes it very useful for mapping cortical regions related to auditory processing and understanding structural changes after implantation [109]. However, fMRI has lower temporal resolution compared to EEG and is more expensive, which may limit its frequent use in clinical settings. The noise interference of fMRI also complicates auditory evoked experiments, and the requirement for patients to remain stationary for extended periods poses a challenge for pediatric patients.

PET provides insights into the brain's metabolic activity, offering unique information that EEG and fMRI cannot [110]. It is useful for evaluating long‐term changes in brain activity and understanding the metabolic effects of cochlear implants. However, PET involves exposure to radioactive tracers, making it less suitable for repeated use, especially in children. Its spatial and temporal resolutions are also lower compared to fMRI and EEG.

MEG combines high temporal resolution with relatively high spatial resolution, similar to EEG. It helps locate neural activity and understand functional connections in the brain [111]. However, MEG is expensive, requires specialized equipment, and is sensitive to external magnetic interference, which limits its accessibility and application in routine clinical practice.

NIRS is a non‐invasive method suitable for long‐term monitoring and is not highly sensitive to motion artifacts [112], making it ideal for pediatric patients. It measures changes in blood oxygenation, providing insights into cortical activity. However, its spatial resolution is low and limited to monitoring surface cortical activity [113].

In summary, each neuroimaging technique has advantages and limitations, as shown in Table 3. To address these limitations, researchers often combine multiple techniques (such as NIRS) for multimodal research to compensate for the shortcomings of a single technique. Additionally, optimizing experimental design, developing noise reduction algorithms, and improving subject experience can enhance the effectiveness and reliability of clinical applications.

**TABLE 3:** Comparative analysis of neuroimaging techniques in auditory cortex studies.

Criteria	EEG	fMRI	PET	MEG	NIRS
Temporal resolution	High (ms)	Low (s)	Moderate (min)	High (ms)	Moderate (s)
Spatial resolution	Low (cm)	High (mm)	Moderate (cm)	High (mm)	Low (cm)
Invasiveness	Non‐invasive	Non‐invasive	Minimally invasive (radioactive tracers)	Non‐invasive	Non‐invasive

Cost	Low	High	Very high	High	Moderate
Clinical applications	Real‐time monitoring of neural activity	Precise localization of brain activity	Assessment of metabolic activity	Localizing neural activity, functional connectivity	Monitoring blood oxygenation, surface cortical activity
Patient requirements	Minimal, suitable for children	Requires stillness, challenging for children	Minimal, concerns with repeated use	Requires stillness, sensitive to interference	Minimal, suitable for children
Utility in cochlear implant studies	Effective for rapid changes and neuroplasticity	Valuable for mapping cortical areas and structural changes	Understanding metabolic implications, long‐term changes	Localizing neural activity, functional connectivity	Monitoring blood oxygenation, cortical activity

## 4 Challenge and Future Trends

As ABCI continues to advance, its application in auditory and language‐related studies has shown great promise, particularly for assessing neural plasticity and auditory function in infants and individuals who cannot undergo traditional hearing tests. However, the clinical application still faces several significant challenges. Addressing these challenges and leveraging emerging trends will be crucial for enhancing the effectiveness and reliability of these technologies in clinical settings.

### 4.1 Limitations and Challenges of ABCI in Auditory Assessment

ABCI technology has been extensively studied for objective auditory assessment due to its advantages of objectivity, accuracy, and the lack of need for patient cooperation [33, 102], making it particularly suitable for pediatric patients [81]. However, its limitations must be considered in clinical applications.

1. Signal noise: Auditory evoked potentials are delicate signals, and EEG data is susceptible to interference such as muscle activity, eye movement, and ambient noise. These interferences can obscure the neural signals related to auditory processing, making obtaining accurate and reliable data challenging. To mitigate this issue, developing and implementing advanced signal processing techniques and algorithms to filter out noise and artifacts from EEG recordings is essential. Additionally, conducting EEG recordings in controlled environments can help minimize ambient noise and other interferences, thus enhancing the reliability of the data.
2. Technical threshold: EEG provides valuable information on brain electrical activity, but interpreting these data requires experienced technicians or clinicians. The complexity of EEG data analysis can be a barrier to its widespread clinical application, necessitating specialized training and expertise. Addressing this challenge involves developing comprehensive training programs for clinicians and technicians to enhance their skills in EEG data interpretation. Moreover, designing and implementing user‐friendly software tools that assist in analyzing and interpreting EEG data can make the technology more accessible and easier to use. On the other hand, it can also provide immediate evaluation results for pediatric patients and parents.
3. Limitations in deep structures: EEG primarily measures electrical activity on the surface of the cerebral cortex, limiting its assessment of deeper brain structures [25]. Advanced auditory functions may involve deeper brain regions that EEG does not directly monitor, thus providing an incomplete image of auditory processing. To overcome this limitation, combining EEG with other neuroimaging techniques, such as fMRI and fNIRS, can provide a more comprehensive view of brain activity. Using high‐density EEG systems can also improve spatial resolution and enhance the detection of deeper brain activity [108].
4. Artifacts from hearing devices: When applying ABCI to assess the auditory rehabilitation of CI users, the EEG recording contains electrical artifacts introduced by the CI [114]. Developing algorithms that can effectively extract auditory features from these artifact‐laden recordings remains a significant challenge for the clinical application of ABCI technology. To address this, developing and applying advanced artifact removal algorithms specifically designed to identify and remove artifacts from EEG data related to cochlear implants is necessary. Implementing calibration techniques before recording sessions can also help account for and mitigate the impact of electrical artifacts [115].
5. Brand diversity: Variability exists among CI brands regarding coding strategies, electrode designs, and signal processing algorithms, complicating data interpretation and comparison. Establishing uniform data standardization methods is imperative to overcome these challenges associated with diverse CI brands, ensuring the efficacy and comparability of assessments. This can be achieved by developing and adopting standardization protocols for EEG data collection and analysis across different CI brands. Collaborative research among manufacturers, researchers, and clinicians can foster the creation of unified standards and guidelines.
6. Decoding complex abilities: Analysis of electroencephalographic patterns during the reception of specific auditory stimuli or task performance enables the evaluation of patients' auditory‐related complex abilities, such as attention allocation, auditory memory, and cognitive ability. This yields a more comprehensive understanding of their auditory and language functions, furnishing invaluable insights for tailoring rehabilitation and treatment plans on an individualized basis.
7. Specific challenges in pediatric patients: Pediatric patients face unique challenges in implementing ABCI technology. Infants and young children, in particular, may struggle to remain still and cooperative during EEG recording sessions, leading to motion artifacts and incomplete data that complicate analysis and interpretation. Designing tests that do not require children's attention modulation and involving parents in the testing process can help pediatric patients stay calm and still during EEG recording [63]. Additionally, the neurological responses of pediatric patients can vary greatly depending on age and developmental stage. Younger children may exhibit different auditory processing patterns and neural plasticity compared to older children [43]. Therefore, it is necessary to consider these differences in clinical evaluation and develop age‐specific protocols and standardized data to interpret evaluation results at different developmental stages accurately. Conducting longitudinal studies can also provide valuable insights into the developmental trajectory of auditory processing and neural plasticity in pediatric patients [88, 102]. Furthermore, EEG data from pediatric patients are often more susceptible to artifacts from various sources, such as movement, crying, or anxiety. Effectively handling and reducing these artifacts is crucial for obtaining reliable data. Therefore, developing advanced artifact elimination techniques and algorithms tailored for pediatric EEG data is essential. Additionally, the ethical considerations associated with using ABCI technology in pediatric patients must be addressed. Obtaining informed consent from both the children and their caregivers is crucial. Researchers and clinicians must ensure that the consent process is thorough and that the caregivers fully understand the procedures, potential risks, and benefits involved. Potential discomforts, such as the inconvenience or anxiety associated with the EEG setup, should be minimized through careful planning and creating a reassuring environment for the child.

### 4.2 Future Trends of ABCI Applications

Addressing these challenges will pave the way for more effective and reliable applications of ABCI technology in clinical settings. By implementing the proposed solutions and advancing our understanding of the unique needs of pediatric patients, we can enhance the utility of EEG and related technologies in auditory assessments. Several emerging trends and technological innovations hold promise for further improving the application of ABCI technology.

1. Applications of deep learning and artificial intelligence in data analysis: The advent of deep learning and other AI technologies offers the possibility for analyzing massive, complex EEG data, playing a role in automatic auditory feature recognition, anomaly detection, and result interpretation. This can lead to more precise assessments and personalized treatment plans.
2. Integration of multimodal imaging: Combining EEG technology with other imaging techniques (such as fMRI, fNIRS, etc.) can enhance source localization accuracy and provide a more comprehensive perspective for auditory function research [116]. Multimodal imaging allows researchers to cross‐validate findings from different techniques, thereby increasing the reliability and depth of the data obtained. This integrated approach can offer a more holistic understanding of the brain's response to auditory stimuli and the neural mechanisms underlying auditory processing and rehabilitation.
3. Personalized auditory assessment and intervention: Through EEG assessment, more personalized auditory evaluation results can be offered for each individual, subsequently guiding more precise treatments and interventions. Personalized auditory assessments can take into account the unique neural patterns and auditory processing capabilities of each patient, allowing for tailored rehabilitation strategies that are more effective. This approach can also provide valuable insights for adjusting CI processors and optimizing their performance to meet the specific needs of each user.
4. More in‐depth studies on cognitive and social hearing: Future research will focus more on the interaction of auditory and other cognitive functions (such as attention and memory) and auditory processing in complex social scenarios. Exploring these interactions can provide deeper insights into how auditory information is integrated and processed in the brain, particularly in real‐world environments. Understanding these mechanisms can help develop interventions that improve auditory function and cognitive and social outcomes for individuals with hearing impairments.
5. Expansion of telehealth and remote monitoring: Integrating ABCI technology with telehealth platforms can enable remote monitoring and assessment of auditory function. Remote monitoring can also support continuous assessment and adjustment of rehabilitation programs, ensuring that patients receive optimal care without frequent in‐person visits.
6. Broader clinical applications: ABCI assessment may be increasingly used as a diagnostic and evaluation tool for various auditory disorders and related diseases. The broader application of ABCI in clinical settings can enhance the assessment and management of conditions such as auditory processing disorder, tinnitus, and auditory neuropathy. As ABCI continues to advance, its utility in diverse auditory scenarios will expand, providing clinicians with a powerful tool for improving patient outcomes.

## 5 Conclusion

In conclusion, EEG presents a distinctive and reliable instrument for objectively evaluating auditory abilities in children with CIs. The clinical applicability of EEG is abundant due to the benefits of being non‐invasive, sensitive, cost‐effective, and flexible. This review has offered an in‐depth analysis of the reconstruction of auditory function post‐cochlear implantation on multiple fronts, encompassing the understanding of sound's physical properties, non‐linguistic music perception, and language comprehension. Emphasis has been placed on the biomarkers of auditory rehabilitation in children with CIs and post‐implantation cortical reorganization, underscoring the variances in auditory feedback between individuals lacking hearing and those possessing normal auditory functions. To conclude, the evolution of efficient auditory feature extraction algorithms and the multimodal fusion of EEG is expected to augment the precision and applicability of objective auditory assessments, thus furnishing a more accurate basis for clinical auditory interventions.

## Conflicts of Interest

The authors declare no conflicts of interest.
